# Comparison of the Body Composition of Caucasian Young Normal Body Mass Women, Measured in the Follicular Phase, Depending on the Carbohydrate Diet Level

**DOI:** 10.3390/medicina54060104

**Published:** 2018-12-05

**Authors:** Dominika Głąbska, Karolina Cackowska, Dominika Guzek

**Affiliations:** 1 Department of Dietetics, Faculty of Human Nutrition and Consumer Sciences, Warsaw University of Life Sciences (WULS-SGGW), 159c Nowoursynowska Str., 02-776 Warsaw, Poland; karolina_cackowska@sggw.pl; 2 Department of Organization and Consumption Economics, Faculty of Human Nutrition and Consumer Sciences, Warsaw University of Life Sciences (WULS-SGGW), 159c Nowoursynowska Str., 02-776 Warsaw, Poland; dominika_guzek@sggw.pl

**Keywords:** body composition, bioelectrical impedance, reproducibility, carbohydrate intake, water-electrolyte balance, extracellular water content, intracellular water content

## Abstract

*Background and objectives*: Some publications indicate the possibility of the influence of meal nutritional value on results of bioelectrical impedance, and of the relation between the long-term carbohydrate intake and body composition. The aim of the presented study was to evaluate the influence of long-term intake of carbohydrates on body composition results assessed using the bioelectrical impedance of Caucasian young women with normal body mass, who were in the follicular phase of their menstrual cycle. *Materials and Methods*: Body composition was assessed in 100 women (18–30 years), according to strict rules, to minimize the influence of disturbing factors and by using two types of bioelectrical impedance device of the same operator to eliminate the influence of measurement (BIA 101/SC and BIA 101/ASE by Akern Srl, Firenze, Italy with the Bodygram 1.31 software and its equations by Akern Srl, Firenze, Italy). The analysis included validation of reproducibility of body composition assessment (fat, fat-free, body cell and muscle mass, water, extracellular water, and intracellular water content), and comparison of body composition for groups characterized by carbohydrate content <50% (*n* = 55) and >50% of the energy value of the diet (*n* = 45). *Results*: Analysis conducted using Bland–Altman method, analysis of correlation, analysis of quartile distribution, and weighted κ statistic revealed a positively validated reproducibility, but extracellular water associations were the weakest. Depending on the device, participants characterized by higher carbohydrate intake had significantly higher intracellular water content (*p* = 0.0448), or close to significantly higher (*p* = 0.0851) than those characterized by lower carbohydrate intake, whose extracellular water content was close to significantly lower (*p* = 0.0638) or did not differ. *Conclusions*: The long-term, moderately reduced, carbohydrate intake may cause the shift of intracellular water to the extracellular space and, as a result, influence the body composition results.

## 1. Introduction

The bioelectrical impedance enables the assessment of body composition and it is indicated as a method that allows the determination of both water and tissues content in individuals without serious fluid and electrolyte disturbances [1]. Bioelectrical impedance is a valid method in large epidemiological studies, as the results of measurements are significantly associated with the health outcomes and it could be a better predictor of the risk of chronic diseases, than body mass index (BMI) [2], while they are equally good as BMI in detecting obesity [3]. At the same time, due to the influence of an assessed population, specific equations must be always used to predict the body composition [4] and must be also validated for chronic diseases common in the groups [5].

As the frequency of applying bioelectrical impedance is observed to be growing worldwide, it was necessary to elaborate the detailed recommendations of the measurement procedure in clinical applications [6]. However, the food and drink consumption before the measurement is among such factors for which the influence is not clearly defined. Kyle et al. [6] recommended fasting with no alcohol for over 8 h before the measurement, but at the same time they indicated that shorter periods of fasting may be acceptable in the clinical practice. Simultaneously, Dixon et al. [7] indicated that 20 min after meal the results of the measurement are significantly different than those observed in a fasting state, which was also observed by Gallagher et al. [8] for 5 h after meal. Above and beyond, the study of Slinde and Rossander-Hulthén [9] showed that not only the change of the measured impedance values lasts 2–4 h after each meal, but also during the day, the observed change is additive. However, González-Correa & Caicedo-Eraso [10], based on the study of Gonzalez et al. [11], showed that conducting the measurement 2 h after a meal contributes to 2.9% of the measurement error only, which corresponds with the recommendations of Kyle et al. [6], indicating the shorter periods of fasting as acceptable in clinical practice.

However, the recent publications indicate the possibility of the influence of the nutritional value of a consumed meal and macronutrients proportions, including the influence of carbohydrates intake. Androutsos et al. [12], found that in spite of the fact, that the change of the bioelectrical impedance results is observed for over 2 h, regardless of the type of the meal, for a high-carbohydrate meal, it is observed earlier, than for a high-fat meal. Similarly, in the studies of Dixon et al. [13,14], the carbohydrate/electrolyte drink contributed to changes of body fat mass and body water content; however, the observed changes were attributed to the content of water in the consumed beverage. In the context of the carbohydrate content in the diet, it must also be indicated that the changes of body composition after a hypocaloric diet are associated with the carbohydrate content in the diet [15]. It may be supposed that the carbohydrate intake may contribute to the changes of body composition not only due to the effect of diet, but also due to the long-term effect of carbohydrate intake changing the bioelectrical impedance results.

The aim of the presented study was to evaluate the influence of long-term intake of carbohydrates on body composition results assessed using the bioelectrical impedance of Caucasian young women with normal body mass, who were in the follicular phase of their menstrual cycle.

## 2. Materials and Methods

The study was conducted according to the guidelines of the Declaration of Helsinki, and all procedures involving human subjects were approved by the Bioethical Commission of the National Food and Nutrition Institute in Warsaw (no. 0701/2015).

### 2.1. Recruitment of Participants and Inclusion Criteria

The invitation to participate in the study as well as information about the inclusion criteria were distributed via social media. In order to obtain the reliable estimation of the typical carbohydrate intake, the recruitment was conducted only among female students and graduates of the nutritional faculties of the Warsaw University of Life Sciences (faculties of dietetics, human nutrition, and food evaluation), characterized by the nutritional knowledge that enable to conduct a reliable dietary record on a typical day (purposive sampling). The recruitment for the study was done exclusively for the presented analysis and no additional dietary intervention or other biochemical analysis was conducted.

The inclusion criteria were as follows: Caucasian women, aged 18–30 years, characterized by BMI ∈ (18.5–25.0 kg/m^2^), stable body mass, without any metabolic disorders or other chronic diseases, and living in Warsaw. The exclusion criteria were as follows: women with irregular menstrual cycles, with amenorrhea, applying hormonal contraception, with epilepsy, having pacemakers or other simulators implanted, having orthopedic prosthesis or other metal implants, having abnormal body, limb or trunk build (e.g., after serious surgical procedures and resections, after limb amputations, with serious scoliosis), women professionally practicing sports, pregnant, lactating, undergoing body mass reduction, and on any special diet.

The BMI was calculated based on the measured weight and height, while the measurement was conducted using an electronic medical weighing scale with a stadiometer. The measurement was conducted on the basis of widely accepted and applied rules, while the height was measured with an accuracy of 0.5 cm and the weight—with an accuracy of 0.1 kg.

From the group of individuals who volunteered to participate in the study, met the inclusion criteria and not excluded due to the exclusion criteria, finally, 100 women took part in the study after providing a written consent to participate. The participating women, aged 18–29 years (22.70 ± 2.12 years; median of 23.0 years), were characterized by a BMI of 20.83 ± 1.71 kg/m^2^ (median of 20.54 kg/m^2^; varied from 18.53 to 24.96 kg/m^2^).

### 2.2. Study Design

The study was conducted in the Dietary Outpatient Clinic of the Faculty of Human Nutrition and Consumer Sciences, Warsaw University of Life Sciences. Due to the need to conduct the measurements of bioelectrical impedance for assessing the body composition in the follicular phase and a specific part of the menstrual cycle (6–11 days), the measurements were conducted for three months, during the autumn period, after a previous day arrangement confirmation.

The measurements were conducted according to very strict rules, in order to minimize the influence of disturbing factors on the measurement errors. In order to eliminate the influence of measurement and operator, all the measurements were conducted twice (using two types of bioelectrical impedance device), and by the same operator, as is commonly applied [16].

The obtained results were compared between sub-group characterized by the carbohydrate content lower than 50% of the energy value of the diet (*n* = 55), and by carbohydrate content higher than 50% of the energy value of the diet (*n* = 45).

#### 2.2.1. Preparation for the Measurement

After the qualification to the study, each participant was informed about the requirements of the necessary preparation for the measurement. It included information that the measurement must be conducted after menstruation, between 6th and 11th day of the menstrual cycle (follicular phase of the menstrual cycle), which is commonly applied [17,18], in order to avoid the influence of the menstrual cycle on the results of the body composition [19]. For the conducted study, standardizing the phase of the menstrual cycle was especially important, as it is proven that also the phase influences the carbohydrate intake [20].

Moreover, it was specified that the measurement must be conducted after a day when participants are characterized with a typical physical activity and a typical diet. The information included the detailed recommendations associated with the preceding day preparation for the measurement and the recommendations for the measurement day.

Among preceding day recommendations, it was indicated that participants should not consume any alcoholic beverage, coffee, and other caffeine beverage as well as should avoid any excessive physical activity (such as sport practicing or training). Participants were also informed that they should prepare a 24-h dietary record of all food products consumed and beverages drunk on the preceding day.

Among the measurement day recommendations, it was indicated that participants should have the measurement conducted in a fasting state (last meal consumed should be at least 8 h before the measurement), should not drink before the measurement (last beverage drunk should be at least 8 h before the measurement), should urinate 30 min before the measurement and defecate on the day of measurement—before the measurement.

#### 2.2.2. Dietary Record

The diet assessment was conducted based on self-reported data, declared in the 24-h dietary record of all food products consumed and beverages drunk, conducted on the preceding day. To provide the reliable estimates of the intake, all participants received a detailed instruction on the principles of making the dietary record, as well as on the necessity of an accurate and scrupulous recording of all food products consumed and beverages drunk. Participants were instructed to conduct the record on the day before the measurement day, which should also be a day when their diet is typical.

Due to the fact that the purposive sampling was applied, the recruited participants were characterized by the general nutritional knowledge (female students of faculties of dietetics, human nutrition, and food evaluation). They were asked to conduct the 24-h dietary record during a day when their diet is typical, in order to assess the carbohydrate level in their everyday diet. In general, among factors contributing to unreliable dietary intake reporting may be those associated with lack of nutritional knowledge (e.g., lack of knowledge of the composition of mixed dishes, inability to estimate portion sizes accurately) [21]. Taking it into account, for the assessed group, characterized by the general nutritional knowledge the diet recorded as typical, was interpreted as credible. Such approach is presented also by other authors who assess only 24-h intake for the assessment of the typical nutrients (carbohydrates) intake in the diet [22].

The dietary record was conducted based on widely accepted and applied rules—using a structured format, with additional questions about the name of the meal, time and location of consumption, meal ingredients, and the weight (weighed using a kitchen scale) or the size of the serving (estimated using standard household measures) [23].

The sizes of servings were verified and re-calculated per weight of the serving, by a dietitian, using the Polish food model booklet [24]. The carbohydrate intake in diets was analyzed using the Polish dietician software—Dieta 5.0 (National Food and Nutrition Institute, Warsaw, Poland, 2011) and the Polish database of the nutritional value of products [25].

Based on the assessed carbohydrate intake in a typical diet, during the analysis of the results, women were divided into two groups—characterized by the carbohydrate content lower than 50% of the energy value of the diet (*n* = 55), and by carbohydrate content higher than 50% of the energy value of the diet (*n* = 45). The BMI was compared and no significant differences (*p* > 0.05) were observed between participants characterized by the carbohydrate content lower than 50% of the energy value of the diet (median of BMI—21.0 kg/m^2^, differing from 18.5 kg/m^2^ to 24.7 kg/m^2^), and by carbohydrate content higher than 50% (median of BMI—20.4 kg/m^2^, differing from 18.5 kg/m^2^ to 25.0 kg/m^2^).

#### 2.2.3. Bioelectrical Impedance Measurement

Due to the need to conduct the measurements in the fasting state, the measurements were conducted in the morning. Directly before the measurement, participants were asked to take off their shoes and clothes (the measurements were conducted while they were in the light underwear—in panties and a no wire bra), without metal elements in the underwear, and without jewelry.

The measurement was conducted in a supine recumbent position, while arms were separated from the trunk by about 30° and legs—separated by about 45°, as recommended [6]. In order to obtain such a position and to avoid the contact of body with metal elements of the medical couch, the measurements were conducted on two layers of a polyurethane foam matte and a fabric matte isolating from the floor without any metal or conductive elements. Before the measurement, participants stayed for 5 min in the supine recumbent position.

Directly before the measurement, the dorsal part of the right hand and the dorsal part of the right feet were rubbed using medical disinfection cotton pads and when the surface was dried, the electrodes were placed. In the dorsal part of both hand and feet, two standard Ag-AgCl rectangular Pro-Tab, PT 2334, Bio Protech electrodes (contact area higher than 4 cm^2^) were placed (tetrapolar electrode configuration), with a distance between them of at least 5 cm, without any skin lesions at the location of the electrodes [26].

In order to conduct the assessment of the reproducibility, the two repeated measurements were conducted using two types of bioelectrical impedance devices, which conduct the measurement with a frequency of 50 kHz, obtained from the same producer: BIA 101/SC (Akern Srl, Firenze, Italy) and BIA 101/ASE (Akern Srl, Firenze, Italy), with 5 min interval between measurements. A similar approach was applied by Ramírez-Vélez et al. [27] for devices from another producer. The measurements were conducted in a random order. The data of resistance and reactance were recorded, while they remained stable. As the producer of BIA 101 recommends to use the Bodygram software and not other predictive equations while the results of impedance may be specific for the device, the obtained resistance and reactance data were re-calculated using the Bodygram 1.31 and its equations (Akern Srl, Firenze, Italy), into fat mass (% of body mass), fat-free mass (% of body mass), body cell mass (% of body mass), muscle mass (% of body mass), water content (% of body mass), extracellular water content (% of water content), and intracellular water content (% of water content).

### 2.3. Statistical Analysis

The conducted analysis included two steps:

(1) validation of the reproducibility of obtained data of the body composition assessment (fat mass, fat-free mass, body cell mass, muscle mass, water content, extracellular water content, and intracellular water content) conducted using two types of bioelectrical impedance devices,
(2) comparison of the data of the body composition assessment conducted using the bioelectrical impedance, obtained for groups of participants characterized by carbohydrate content lower than 50% of the energy value of the diet (*n* = 55), and higher than 50% of the energy value of the diet (*n* = 45).

The validation of the reproducibility of the obtained data (the 1st step of the statistical analysis) was conducted according to the same methodology, as in the previous studies [28,29]. It included:

(1) Analysis of the Bland–Altman plots—a Bland–Altman index ≤5% (attributed to 95% of individuals observed to be within the LOA) was interpreted as a positive validation of the method [30], while a Bland–Altman index ≤10% (attributed to 90% of individuals observed to be within the LOA) was interpreted as a borderline significant [31].
(2) Analysis of the correlations between results conducted using Pearson correlation (for the parametric distribution) or Spearman’s rank correlation (for the nonparametric distribution), while the distribution was assessed using the Shapiro-Wilk test.
(3) Analysis of the quartiles cross-classification.
(4) Analysis of the weighted κ statistic with linear weighting for quartiles cross-classification—values lower than 0.20 were interpreted as slight agreement, 0.21–0.40—fair, 0.41–0.60—moderate, 0.61–0.80—substantial, and 0.81–1.0—almost perfect agreement [32].

The comparison of the data of the body composition assessment conducted for groups of participants characterized by various carbohydrate content (the second step of the statistical analysis) was conducted after a verification of the normality of distribution of the results, performed using the Shapiro–Wilk test. Afterwards, for the parametric distribution mean accompanied by Standard Deviation (SD) values were presented and Student’s *t*-test was applied, while for the nonparametric distribution median accompanied by minimum and maximum values were presented and Mann–Whitney U test was applied.

The *p* ≤ 0.05 was indicated as significant. Statistical analysis was performed using Statistica 8.0 (StatSoft Inc., Tulsa, OK, USA) and Bland–Altman Statistica macro by Matt Coates 2009 (StatSoft Inc., Tulsa, OK, USA).

## 3. Results

The analysis of the obtained data conducted using the Bland–Altman method revealed positively validated reproducibility for fat mass, fat-free mass, body cell mass, muscle mass, water content, and intracellular water content. For extracellular water content, the Bland–Altman index indicated borderline reproducibility.

**Figure 1:**
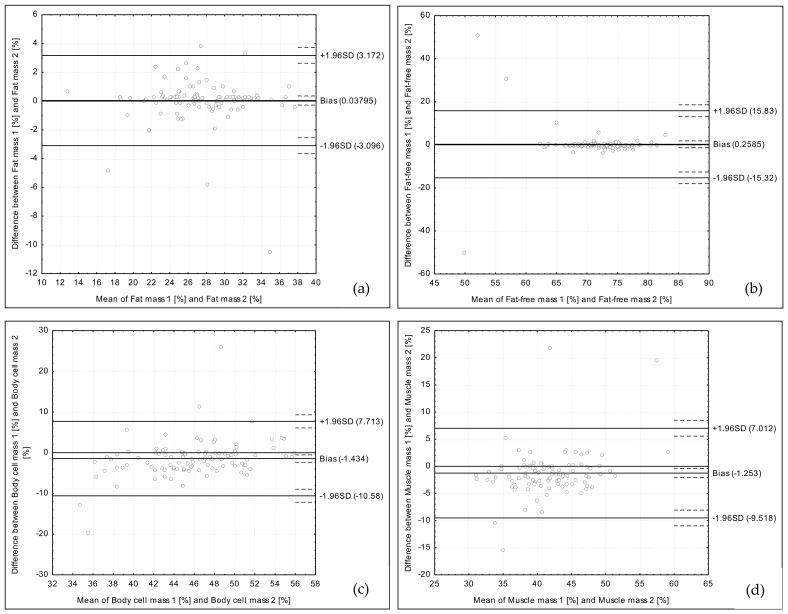
Bland–Altman plots comparing fat mass (**a**), fat-free mass (**b**), body cell mass (**c**), and muscle mass (**d**) assessed using two types of the bioelectrical impedance devices.

The Bland–Altman plot for fat mass assessed using two bioelectrical impedance devices is presented in Figure 1a. The 95 out of 100 individuals were within the LOA value (the Bland–Altman index of 5.0%).

The Bland–Altman plot for fat-free mass assessed using two bioelectrical impedance devices is presented in Figure 1b. The 97 out of 100 individuals were within the LOA value (the Bland–Altman index of 3.0%).

The Bland–Altman plot for body cell mass assessed using two bioelectrical impedance devices is presented in Figure 1c. The 95 out of 100 individuals were within the LOA value (the Bland–Altman index of 5.0%).

The Bland–Altman plot for muscle mass assessed using two bioelectrical impedance devices is presented in Figure 1d. The 96 out of 100 individuals were within the LOA value (the Bland–Altman index of 4.0%).

**Figure 2:**
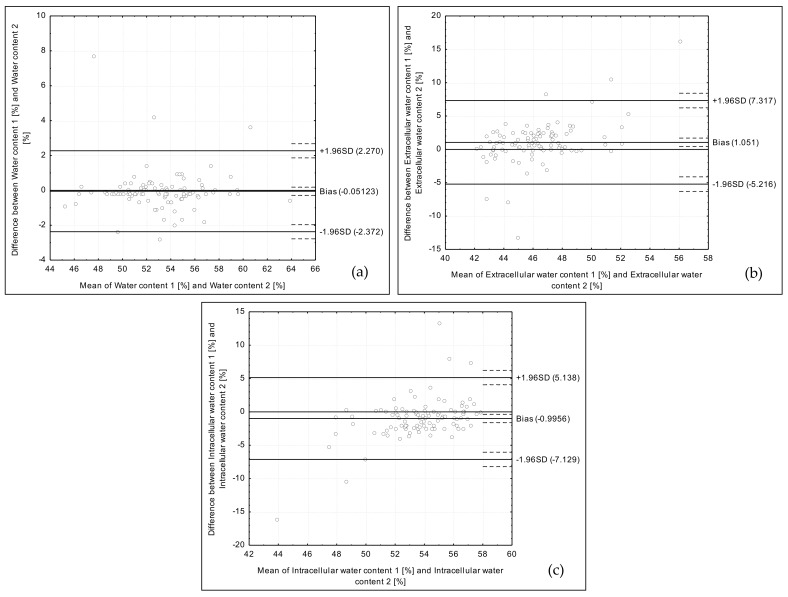
Bland–Altman plots comparing water content (**a**), extracellular water content (**b**), and intracellular water content (**c**) assessed using two types of the bioelectrical impedance devices.

The Bland–Altman plot for water content assessed using two bioelectrical impedance devices is presented in Figure 2a. The 95 out of 100 individuals were within the LOA value (the Bland–Altman index of 5.0%).

The Bland–Altman plot for extracellular water content assessed using two bioelectrical impedance devices is presented in Figure 2b. The 94 out of 100 individuals were within the LOA value (the Bland–Altman index of 6.0%).

The Bland–Altman plot for intracellular water content assessed using two bioelectrical impedance devices is presented in Figure 2c. The 95 out of 100 individuals were within the LOA value (the Bland–Altman index of 5.0%).

The additional methods of statistical analysis confirmed the observed positively validated reproducibility, which is presented in Table 1. The analysis of correlation indicated positive, moderate to perfect, linear relationship between the reproduced measurements. The lowest correlation coefficient was observed for the extracellular water content, while the highest correlation coefficient was observed for the fat mass. Similarly, in the analysis of the quartile distribution, the share of grossly misclassified results was not higher than 4% (observed for extracellular water content). Moreover, according to the criteria of Landis & Koch [32], the weighted κ statistics were interpreted as moderate (for extracellular water content, intracellular water content, and body cell mass), substantial (for muscle mass and fat-free mass) or almost perfect agreement (for fat mass and water content).

The comparison of the body composition assessed using BIA 101/SC (Akern Srl, Firenze, Italy) in groups of females declaring the typical diet characterized by carbohydrate content lower than 50% of energy value of diet and declaring the typical diet characterized by carbohydrate content higher than 50% of energy value of diet is presented in Table 2. The difference close to the significance was observed for intracellular water content (*p* = 0.0851), as the higher intracellular water content (median of 54.8%) was observed for women characterized by carbohydrate content higher than 50% of the energy value of the diet, than for women characterized by carbohydrate content lower than 50% of the energy value of the diet (median of 53.9%).

The comparison of the body composition assessed using BIA 101/ASE (Akern Srl, Firenze, Italy) in groups of females, who declared the typical diet characterized by carbohydrate content lower than 50% of the energy value of the diet and by carbohydrate content higher than 50% of the energy value of the diet is presented in Table 3. The statistically significant difference was observed for intracellular water content (*p* = 0.0448), as higher intracellular water content (median of 53.6%) was observed for women characterized by carbohydrate content higher than 50% of the energy value of the diet, than for women characterized by carbohydrate content lower than 50% of the energy value of the diet (median of 52.9%). At the same time, the difference close to significance was observed for extracellular water content (*p* = 0.0638), as lower extracellular water content (median of 46.4%) was observed for women characterized by carbohydrate content higher than 50% of the energy value of the diet, than for women characterized by carbohydrate content lower than 50% of the energy value of the diet (median of 47.1%).

## 4. Discussion

### 4.1. Carbohydrate Intake Level

The main factors that may influence the results of body composition assessment are not only those factors that are included into equations to calculate the body composition, such as anthropometric measurements, gender, age, and ethnic group [33], but also the measurement procedure, used electrodes, and measurement errors [10,33]. However, for many additional factors, such as dietary intake of specific nutrients on the day leading up the day of measurement, the influence so far was not fully analyzed and precisely defined yet. Also, the number of recommendations for the bioelectrical impedance measurement associated with food and drink consumption is limited—they are mainly concerned about the fasting state and the intake of products such as alcoholic beverages, coffee, tea, or chocolate [6,10].

The conducted statistical analysis revealed that a typical carbohydrate intake may influence the observed results of the body composition, assessed using the bioelectrical impedance measurement. The lower carbohydrate intake may contribute to lower intracellular water content and higher extracellular water content than for higher carbohydrate intake, but without any difference in total water content. The observed results may suggest the shift of intracellular water to the extracellular space, due to low carbohydrate intake.

It is well known, that the generally recommended carbohydrate intake is higher than 50% of the energy value of the diet, as the World Health Organization indicates that a diet should contain at least 55% of total energy value from various carbohydrates [34]. But, at the same time, the carbohydrate content of 40–65% of the energy value of the diet is classified as a moderate carbohydrate intake [35]. As a result, the group of individuals in own study characterized by a carbohydrate intake lower than 50% of the energy value of the diet may be classified as young female individuals with proper body mass, applying the moderately reduced carbohydrate share, normocaloric diet. It must be emphasized, that the assessed diet must be treated as a moderately reduced carbohydrate diet, as some studies assess the very-low-carbohydrate ketogenic diet, characterized by 10% of the energy value of the diet from carbohydrate [36], being incomparable with the carbohydrate intake lower than 50% of the energy value of the diet.

The moderate carbohydrate reduction observed in own study must be analyzed, considering the fact that the health effects of the carbohydrate reduction are related to the level of the reduction. From the systematic review and meta-analysis of the observational studies, by Noto et al. [37], it was stated that low-carbohydrate diets are associated with a significantly increased all-cause mortality risk. However, it was not stated for a moderately reduced carbohydrate share, similarly, as in the study of Nilsson et al. [38] where moderate carbohydrate intake reduction was not related to the elevated cancer risk. At the same time, low-carbohydrate diets were observed to improve the glycemic response and to reduce the body mass in overweight and obese individuals, which was not observed for moderate-carbohydrate diets [39].

### 4.2. Water Content Changes

While the water content changes associated with applied diet are mentioned, the rapid initial water loss is the well-known process, which contributes to the rapid initial body mass loss on a low-calorie diet [40]. Similarly, in the case of individuals on a ketogenic diet the water loss is commonly stated [41], and it is associated with the fact that patients urinate more frequently because carbohydrates promote water retention. It results from two mechanisms. Firstly, a higher carbohydrate concentration decreases the rate of gastric emptying [42], causing slower fluid movement into the bloodstream that attenuates the urine production [43]. Secondly, higher carbohydrate content may stimulate higher water retention, which is related to glycogen storage, as it is estimated that each 1 g of glycogen is bound to 2.7–4.0 g of water [44]. Inversely, lower carbohydrate intake may increase the urine production, as well as decrease water bounding.

However, in the conducted study, neither low-caloric or ketogenic diet was applied, nor the water loss was observed. Although based on the meta-analysis of Martinoli et al. [45], some overestimation of the measured water content could be assumed, the difference of the body water content between groups would be observed, if the carbohydrate content contributes to the total water loss. As a result, it must be emphasized that not the total body water loss, but only the water compartment shift on a moderately reduced carbohydrate diet was stated.

### 4.3. Role of Water-Electrolyte Balance

The influence of diet on the water compartments was also observed in the study of Marken Lichtenbelt & Fogelholm [46], while a three-month weight-reduction program, including a very-low energy diet (not described in details), applied in a group of young overweight women, was followed by a nine-month weight maintenance program. In the mentioned study, it was observed that after 12 months of the applied program, the extracellular water content was significantly higher than before (increased by 1 kg), similarly, as extracellular water content to intracellular water content ratio also increased to 0.87 ± 0.12 from the baseline 0.78 ± 0.10, while the total body water did not change [47]. The effect observed in the own study may be similar, to the effect from the study of Marken Lichtenbelt & Fogelholm [46] and it may be attributed to the decreased carbohydrate intake commonly applied in the very-low energy diet and stated to be lower than 50% of the energy value of the diet in the own study.

The association between the reduced carbohydrate intake and body water compartments may be explained by the complex associations that regulate insulin and water-electrolyte homeostasis. In normal conditions, low carbohydrate intake is associated with lower blood insulin level, than for the higher carbohydrate intake [47]. Hyperinsulinemia would enhance the renal sodium re-absorption, increasing the sodium level [48,49], while lower blood insulin level causes the increase of the sodium excretion. The study of Rabast et al. [50] confirmed that two types of low-carbohydrate reducing diets applied in obese individuals contributed to higher urinary sodium excretion, than for high-carbohydrate diet. The observed association was explained by Rabast et al. [50] by the effect of elevated glucagon level and the appearance of ketones that both appear in the starving condition and on a low-carbohydrate diet [51].

Afterwards, the decreased extracellular sodium level may force the transport of ions from the cell to the extracellular space, in order to obtain the ion potential equilibrium. Among mechanisms engaged in balancing the equilibrium, in the case of low blood sodium level, are indicated various membrane transporters, including sodium-potassium pump and other possible mechanisms [52]. The need to balance the equilibrium is associated with the mechanisms of extracellular and intracellular compartment homeostasis that favor the maintenance of osmolarity of the extracellular fluid. It results from the fact that the active pumping sodium out from the cell must help to prevent the cellular edema and cell damage [53]. It was also indicated by Kurbel [54] that was based on the study of Baumgarten & Feher [55], it was demonstrated that the only way for the cell to reach the osmotic equilibrium state is to modify the volume until the intracellular ion concentration is balanced by the extracellular ion concentration. It is the first stage, afterward, as the osmotic equilibrium state is obtained, the cell volume becomes the priority, and the accumulation of sodium ions outside the cell draws water out of the cell [56].

The other potential mechanism of the water compartment shift from the cell to the extracellular space may be explained by the results of the study of Shiose et al. [57], as a high-carbohydrate diet, containing 12 g of carbohydrates/kg of body mass contributed to the increase of intracellular water content. It was attributed to the increase of muscle glycogen concentration, which is observed after a carbohydrate load, while it is estimated that glycogen is bound to water at a range of up to 400% of its mass [57]. It is confirmed by general results indicating the effect of glycogen loading on the total body water content measured for athletes [58] and resulting improvement of obtained sport results [59]. Inversely, low carbohydrate intake may contribute to the lower water muscle bounding and, as a result, to lower intracellular water content.

The shift of intracellular water to the extracellular space due to lower carbohydrate intake, supposed in own study, may have an influence on general well-being. It may increase the dehydration vulnerability, because, during moderate dehydration, water is lost initially from the extracellular water compartment [60], which contributes to the decrease of the sport performance [61]. Carbohydrate intake assessment should not only be conducted in the case of athletes, but it should also be included into the bioelectrical impedance measurement. Moreover, as in the analyzed group, the carbohydrate intake was moderately reduced, there is a need to verify the observed associations in a group characterized with a lower carbohydrate share in their diet.

### 4.4. Limitations of the Study

The study was conducted in a homogenous group of young female respondents being in the follicular phase and a specific part of the menstrual cycle (6–11 day). However, there are also specific limitations of the presented study. The main is associated with the fact, that in general diet declared by respondents, independently from the applied method of assessment, may differ from their actual nutritional habits. Especially for the short record periods, such risk may be important. Taking it into account, the further analysis should be conducted in larger groups with strictly controlled diets.

Moreover, the body composition assessment based on the bioelectrical impedance method allows in fact to assess the resistance and reactance, while the other parameters are being predicted using the specific equations. The measurement itself may be a source of errors, but the applied equations are the additional source of errors, while the possibility to predict the water compartments share is also disputable. Taking it into account, the further analysis are recommended to be conducted using the bioimpedance spectroscopy or multi-frequency bioimpedance, being especially valuable for the assessment of fluid distribution.

## 5. Conclusions

Independently from the applied bioelectrical impedance device, the influence of long-term carbohydrate intake on the results of body composition, was observed in Caucasian young normal body mass women in the follicular phase of their menstrual cycle. The long-term, moderately reduced, carbohydrate intake may cause the shift of intracellular water to the extracellular space. The long-term carbohydrate intake may influence the water–electrolyte balance and, as a result, influence the body composition results.

## Figures and Tables

**Table 1:** Comparison of the body composition assessed using two types of the bioelectrical impedance devices, conducted applying analysis of correlation, analysis of quartile distribution and weighted κ statistic.

	Analysis of Correlation		Analysis of Quartile Distribution (%)			Weighted κ Statistic
	*p*-Value	R	The Same Quartile	The Adjacent Quartiles	The Opposite Quartiles	
Fat mass	<0.0001 *	0.9379	81	18	0	0.840
Fat-free mass	<0.0001 **	0.8648	72	25	1	0.744
Body cell mass	<0.0001 *	0.6413	55	38	2	0.568
Muscle mass	<0.0001 **	0.8047	61	35	1	0.648
Water content	<0.0001 *	0.9367	81	18	0	0.840
Extracellular water content	<0.0001 **	0.5954	50	38	4	0.472
Intracellular water content	<0.0001 **	0.6394	49	41	3	0.488

* analysis of correlation conducted using Pearson correlation (for the parametric distribution). ** analysis of correlation conducted using Spearman’s rank correlation (for the nonparametric distribution).

**Table 2:** Comparison of the body composition assessed using BIA 101/SC (Akern Srl, Firenze, Italy) in groups of females declaring the typical diet characterized by carbohydrate content <50% of the energy value of the diet and declaring the typical diet characterized by carbohydrate content >50% of the energy value of the diet.

	Carbohydrate Content <50% of Energy Value of Diet, *n* = 55	Carbohydrate Content >50% of Energy Value of diet, *n* = 45	*p*-Value **
Rz (Ω)	669.0 ± 56.1	681.9 ± 63.8	0.3033
X_c_ (Ω)	73.0 * (20.0–93.0)	76.0 (46.0–98.0)	0.1400
Fat mass (%)	27.4 ± 5.0	27.4 ± 4.1	0.9632
Fat-free mass (%)	73.0 * (26.6–87.6)	72.7 (63.5–81.6)	0.8164
Body cell mass (%)	46.5 ± 4.6	46.6 ± 4.6	0.8924
Muscle mass (%)	42.3 ± 5.2	42.1 ± 4.5	0.8614
Water content (%)	53.2 ± 3.6	53.11 ± 3.0	0.9472
Extracellular water content (%)	46.0 (41.8–50.6)	45.1 * (42.1–51.7)	0.1766
Intracellular water content (%)	53.9 (49.4–58.2)	54.8 * (48.3–57.9)	0.0851

Rz—resistance; X_c_—reactance. * distribution different than parametric – median accompanied by minimum and maximum values presented (verified using Shapiro–Wilk test; *p* ≤ 0.05; for parametric distribution—mean accompanied by SD values presented). ** compared using Student *t*-test (for the parametric distribution) and Mann–Whitney U test (for the nonparametric distribution)—the relevant data are presented based on distribution.

**Table 3:** Comparison of the body composition assessed using BIA 101/ASE (Akern Srl, Firenze, Italy) in groups of females declaring the typical diet characterized by carbohydrate content <50% of the energy value of the diet and declaring the typical diet characterized by carbohydrate content >50% of the energy value of the diet.

	Carbohydrate Content <50% of Energy Value of Diet, *n* = 55	Carbohydrate Content >50% of Energy Value of Diet, *n* = 45	*p*-Value **
Rz (Ω)	665.5 * (588.0–860.0)	674 (570.0–858.0)	0.5051
X_c_ (Ω)	67.0 * (12.3–99.0)	70.0 (53.0–90.7)	0.1812
Fat mass (%)	27.4 ± 4.6	27.5 ± 4.4	0.9274
Fat-free mass (%)	72.8 * (24.9–86.9)	72.3 (62.5–85.2)	0.9862
Body cell mass (%)	44.4 ± 6.5	45.9 ± 5.3	0.2338
Muscle mass (%)	40.7 (27.3–60.4)	40.8 * (31.5–67.2)	0.9227
Water content (%)	53.1 ± 3.4	53.1 ± 3.2	0.9567
Extracellular water content (%)	47.1 * (40.3–64.2)	46.4 (38.3–55.2)	0.0638
Intracellular water content (%)	52.9 * (35.8–59.7)	53.6 (44.8–61.7)	0.0448

Rz—resistance; X_c_—reactance. * distribution different than parametric – median accompanied by minimum and maximum values presented (verified using Shapiro–Wilk test; *p* ≤ 0.05; for parametric distribution—mean accompanied by SD values presented). ** compared using Student *t*-test (for the parametric distribution) and Mann–Whitney U test (for the nonparametric distribution)—the relevant data are presented based on distribution.
